# Multiple Sevoflurane Exposures During the Neonatal Period Cause Hearing Impairment and Loss of Hair Cell Ribbon Synapses in Adult Mice

**DOI:** 10.3389/fnins.2022.945277

**Published:** 2022-07-14

**Authors:** Yufeng Li, Huiqian Yu, Xuehua Zhou, Lin Jin, Wen Li, Geng-Lin Li, Xia Shen

**Affiliations:** ^1^Department of Anesthesiology, Eye & ENT Hospital, Fudan University, Shanghai, China; ^2^ENT Institute and Department of Otorhinolaryngology, Eye & ENT Hospital, Fudan University, Shanghai, China; ^3^State Key Laboratory of Medical Neurobiology and MOE Frontiers Center for Brain Science, Institutes of Brain Science, Fudan University, Shanghai, China

**Keywords:** sevoflurane, hearing impairment, hair cells, ribbon synapse, spiral ganglion neuron nerve fiber, oxidative stress

## Abstract

**Objectives:**

This study aims to investigate the effects of multiple sevoflurane exposures in neonatal mice on hearing function in the later life and explores the underlying mechanisms and protective strategies.

**Materials and Methods:**

Neonatal Kunming mice were exposed to sevoflurane for 3 days. Auditory brainstem response (ABR) and distortion product otoacoustic emission (DPOAE) tests, immunofluorescence, patch-clamp recording, and quantitative real-time PCR were performed to observe hearing function, hair cells, ribbon synapses, nerve fibers, spiral ganglion neurons, and oxidative stress.

**Results:**

Compared to control group, multiple sevoflurane exposures during the neonatal time significantly elevated ABR thresholds at 8 kHz (35.42 ± 1.57 vs. 41.76 ± 1.97 dB, *P* = 0.0256), 16 kHz (23.33 ± 1.28 vs. 33.53 ± 2.523 dB, *P* = 0.0012), 24 kHz (30.00 ± 2.04 vs. 46.76 ± 3.93 dB, *P* = 0.0024), and 32 kHz (41.25 ± 2.31 vs. 54.41 ± 2.94 dB, *P* = 0.0028) on P30, caused ribbon synapse loss on P15 (13.10 ± 0.43 vs. 10.78 ± 0.52, *P* = 0.0039) and P30 (11.24 ± 0.56 vs. 8.50 ± 0.84, *P* = 0.0141), and degenerated spiral ganglion neuron (SGN) nerve fibers on P30 (110.40 ± 16.23 vs. 55.04 ± 8.13, *P* = 0.0073). In addition, the V_*half*_ of calcium current become more negative (−21.99 ± 0.70 vs. −27.17 ± 0.60 mV, *P* < 0.0001), exocytosis was reduced (105.40 ± 19.97 vs. 59.79 ± 10.60 fF, *P* < 0.0001), and *Lpo* was upregulated (*P* = 0.0219) in sevoflurane group than those in control group. *N*-acetylcysteine (NAC) reversed hearing impairment induced by sevoflurane.

**Conclusion:**

The findings suggest that multiple sevoflurane exposures during neonatal time may cause hearing impairment in adult mice. The study also demonstrated that elevated oxidative stress led to ribbon synapses impairment and SGN nerve fibers degeneration, and the interventions of antioxidants alleviated the sevoflurane-induced hearing impairment.

## Introduction

Rapid development in pediatric surgery has resulted in an increased number, complexity, and duration of anesthesia procedures in children. Millions of children are exposed to anesthetics each year during surgical or dental procedures as well as for various imaging procedures, such as magnetic resonance images (MRIs) or computed tomography (CT) scans (Sun et al., 2008), making the impact of anesthesia on children a major health issue of interest among clinicians, parents, and government regulators. Sevoflurane is a volatile anesthetic widely used in pediatric surgery (Wallin et al., 1975). Over the past two decades, numerous animal studies have reported structural and cognitive abnormalities in the brain following exposure to anesthetics, including sevoflurane (Alvarado et al., 2017; Sun et al., 2019). In particular, sevoflurane decreased otoacoustic emissions (OAEs) by ∼2–3 dB, indicating changes in hearing function following anesthesia administration (Gungor et al., 2015).

The developing auditory nervous system is highly fragile and can be impaired by toxic agents (McCartney et al., 1994; Durante et al., 2017). For example, ethanol, an agent that activates gamma-aminobutyric acid (GABA) receptors and depresses *N*-methyl-D-aspartate (NMDA) receptors, exerts detrimental effects on the developing auditory nervous system (Kotch and Sulik, 1992; Church and Kaltenbach, 1997). Considering that currently used general anesthetic agents exert their effect *via* either GABA receptor-enhancing or NMDA receptor-blocking properties, it is plausible that exposure to general anesthetics also has similar ototoxic effects on the developing auditory nervous system. Hearing impairment during the early years of life may result in deficits in language learning, speech, and even intelligence in later life (Hall et al., 2014; Mealings and Harkus, 2020; Sharma et al., 2020). Therefore, concerns about the effects of anesthesia practice on hearing function in children warrant investigation.

Normal hearing requires multiple stages of the sensory signal process in the cochlea and the central nervous system. Along the mammalian auditory pathways, the organ of Corti contains sensory hair cells, including three rows of outer hair cells (OHCs) and one row of inner hair cells (IHCs) (Dallos, 1992), which connect to axonal terminals of spiral ganglion neurons (SGNs) through ribbon synapses in between. In response to sound-induced hair bundle deflection, IHCs respond with a mechanoelectrical transduction (MET) current (Fuchs, 2005; Fettiplace and Hackney, 2006; Richardson et al., 2011) *via* MET channels located in the tips of their stereocilia. This leads to depolarization of hair cells and the opening of voltage-gated Ca^2+^ channels in their basal pole. The resulting Ca^2+^ influx in the active zones triggers the release of synaptic vesicles. Consequently, these processes result in pre-synaptic glutamate neurotransmitter release from IHCs onto SGNs, ultimately activating the auditory pathway.

In our previous study, we reported that *in utero* sevoflurane exposure increases mitochondrial reactive oxygen species stress and decreases autophagy, leading to hearing loss in mice (Yuan et al., 2020). However, whether sevoflurane induces ototoxicity in naïve mice remains unclear. Therefore, the current study aimed to investigate whether exposure to multiple sevoflurane doses exerts toxic effects on the peripheral auditory nervous system and whether it could alter hearing function in the later life of naïve mice.

## Materials and Methods

### Animals

Postnatal day 6 (P6) Kunming mice of both sexes (The Jiesi Jie Laboratory Animal Co., Ltd., Shanghai, China) were used. All animals were housed in quiet rooms with well-controlled humidity and temperature (22–23°C) and 12 h light/dark circadian cycles. Water and mouse chow were provided to animals *ad libitum*.

### Anesthesia Exposure

P6 Kunming mice were randomly assigned to the sevoflurane or control group. In the sevoflurane group, animals were exposed to 3% sevoflurane and 60% oxygen (combined with 40% N_2_) for 2 h daily in an anesthetizing chamber from P6 to P8. In the control group, mice were only exposed to 60% oxygen with 40% N_2_ in the same chamber. A calibrated Vamos side-stream gas analyzer (Dräger, Lübeck, Germany) was used to maintain the concentration of sevoflurane at 3% during the exposure period. The rectal temperature was maintained at 37 ± 0.5°C by keeping a heating pad under the anesthetizing chamber throughout anesthesia exposure. All animals breathed spontaneously during the exposure period.

### Auditory Brainstem Response and Distortion Product Otoacoustic Emission Tests

As previously described (Li et al., 2021), Auditory brainstem response (ABR) tests were conducted at P30. Briefly, mice were anesthetized with 25 mg/kg xylazine and 50 mg/kg esketamine intraperitoneally (i. p.). ABRs were induced with tone-pips of 8, 16, 24, and 32 kHz with descending sound pressure levels and then averaged. The ABR threshold was determined as the lowest sound pressure level at which a typical waveform could be observed. The distortion product otoacoustic emission (DPOAE) tests were conducted at the same frequencies. DPOAE thresholds were determined as the f1 level required to trigger a response at 2f1−f2 above the noise floor.

### *N*-Acetylcysteine Administration

For the intervention studies, the antioxidant agent *N*-acetylcysteine (NAC) (Sigma-Aldrich, St. Louis, MO, United States) was administered i. p. at 20 mg/kg in saline to mice immediately before each of the 3-day sevoflurane exposure sessions. Equivalent volumes of normal saline were administered to mice in the control group.

### RNA Isolation and Quantitative PCR

After being anesthetized with 1.4% isoflurane for 5 min, the mice were euthanized by decapitation. The cochleae were immediately harvested and stored in ice-cold phosphate-buffered saline (PBS) at 0, 6, and 24 h post-anesthesia exposure on P8. Total RNA was extracted using TRIzol (Sigma-Aldrich, St. Louis, MO, United States). Reverse transcription of the total RNA was then carried out using a PrimeScript™ RT Reagent Kit with gDNA Eraser (Perfect Real Time; RR047A; TaKaRa, Dalian, China). PCR quantification was performed with the ABI 7500 real-time PCR system (Applied Biosystems, Foster City, CA, United States) using TB Green^®^ Premix Ex Taq™ (Tli RNaseH Plus) (RR420A; TaKaRa). The forward and reverse primers used were as follows: *Lpo*, F: 5′-CTGGACCAGAAGAGATCCATG-3′, R: 5′-TCACCAGGTGGGAACATGATGG-3′; *xCT*, F: 5′- TGGAGGTCTTTGGTCCTTTG-3′, R: 5′-CCAGGATGTAG CGTCCAAAT-3′; β*-actin*, F: 5′-CCTCTATGCCAACACAGT-3′, R: 5′-AGCCACCAATCCACACAG-3′. β-actin was used to normalize raw data and obtain the relative mRNA expression levels.

### Cochlea Tissue Processing and Immunostaining

At P15 and P30, the inner ears were extracted, and the cochleae were immediately separated and stored in cold PBS. The cochleae were perfused with 4% paraformaldehyde through the opened round and oval windows. The cochleae were fixed for 2 h and decalcified in 10% ethylenediaminetetraacetic acid solution for 1–3 h. For immunofluorescence, only the middle turn of the cochlea was kept; other tissues such as the stria vascularis, spiral ligament, and tectorial membrane were discarded.

After blocking with 10% normal goat serum in 10 mM PBS with 0.3% Triton X-100 for 1 h, the cochleae were incubated for 48 h at 4°C with primary antibodies: rabbit anti-myosin VIIa (1:800; 05012017; Proteus Biosciences, Ramona, CA, United States) for hair cells; mouse IgG1 anti-C-terminal-binding protein-2 (CtBP2; 1:500; 612044; BD Biosciences, CA, United States) for pre-synaptic ribbons in hair cells; mouse IgG2a anti-glutamate receptor 2, extracellular, clone 6C4 (GluA2; 1:2000; MAB 397; Millipore, Schwalbach, Germany) for post-synaptic glutamate receptors; and mouse anti-tubulin β3 (1:800; 801202; Biolegend, CA, United States) for SGNs and nerve fibers innervating hair cells.

The cochleae were washed three times with PBS and then incubated with appropriate Alexa Fluor-conjugated secondary antibodies (1:1000; Jackson Immuno Research, PA, United States) overnight at 4°C.

### Confocal Microscopy Imaging and Quantitative Analysis

Digital images of the cochlea were captured using confocal microscopy (SP8; Leica, Wetzlar, Germany). The resulting confocal images show the three-dimensional structure of the cochlear. The entire z-stack was viewed, rotated, and “resliced” (to view a plane perpendicular to the x–y plane), and the maximum intensity projections of the z-stacks were generated as necessary using the Fiji/ImageJ (NIH, United States).

For quantification of pre-synaptic ribbons and post-synaptic glutamate receptors, z-stack (0.3 μm steps) images were obtained by a 63 × oil immersion objective lens and 2 × digital zoom. The number of CtBP2-positive puncta and GuluA2-positive patches were counted from a specific region, typically six IHCs at afferent nerve fibers, and then divided by the number of IHCs to obtain the number of pre-synaptic ribbons and post-synaptic glutamate receptors per IHC. Ribbon synapses were identified by positive co-localization of double-stained CtBP2 and GluA2. The IHCs and OHCs were also counted in these images.

For the quantification of SGN peripheral nerve fibers, z-stack (1.5 μm steps) images were obtained with a 63× oil immersion objective lens and 2× digital zoom. We converted myosin VIIa and tubulin β-3 images to 8-bit grayscale images and constituted them into a stack using ImageJ. A rectangular area of 150 × 350 pixels around the IHCs was selected for the myosin VIIa images. The density of SGN nerve fibers was expressed as the mean gray value in the chosen area minus the mean gray value the background area of tubulin β−3 images.

For quantification of SGNs, z-stack (2 μm steps) images were obtained using a 40× oil immersion objective lens. A rectangular area of 200 × 300 pixels was outlined, and the SGNs were enumerated using the multipoint tool in Fiji/ImageJ.

### Patch-Clamp Recording

The IHCs of mice on P15 were chosen to carry out the whole-cell patch-clamp recordings. The apical regions of the cochlear were dissected as quickly as possible and immersed in oxygenated extracellular solution containing 125 mM NaCl, 2.8 mM KCl, 1 mM MgCl_2_, 5 mM CaCl_2_, 10 mM HEPES, 2 mM Na-pyruvate, and 5.6 mM d-glucose (290 mOsm, pH 7.40). An upright microscope (Olympus, Tokyo, Japan) with a 60× water-immersion objective was used to visualize the cochlea, and an EPC10/2 amplifier (HEKA, Lambrecht, Germany), driven by PatchMaster software, was used for patch-clamp recordings. Recording pipettes (4–5 MΩ) were pulled from borosilicate glass capillaries (Sutter Instrument, Novato, CA, United States) and coated with dental wax to minimize stray capacitance and enhance Cfast compensation. The internal solution used to fill the recording pipettes contained 135 mM Cs-methane sulfonate, 10 mM CsCl, 10 mM TEA-Cl, 3 Mg-ATP, 10 mM HEPES, 0.5 Na-GTP, and 2 mM EGTA (290 mOsm, pH 7.20).

For measuring calcium current (I_*Ca*_), IHCs were maintained under the voltage clamp mode. Voltage ramps (0.3 s, from −90 mV to +70 mV, holding potential: −90 mV) were applied to record the resulting current. For exocytosis quantification, membrane capacitance measurements were made with the lock-in feature and “Sine + DC” method in PatchMaster (HEKA Electronics, Lambrecht, Germany). The change in capacitance before and after stimulation (△C_*m*_) was used to quantify the exocytosis of synaptic vesicles in IHCs. All patch-clamp experiments were conducted at room temperature. A liquid junction potential of −10 mV was corrected offline.

### Statistical Analysis

Data are shown as mean ± SEM. All data were analyzed with Igor Pro (WaveMetrics, Lake Oswego, OR, United States) and GraphPad^®^ Prism 8. In all experiments, n represents the number of mice, cochleae, or IHCs. Two-tailed unpaired Student’s *t*-tests or Mann–Whitney *U* tests were used for comparison between two groups, except for Figures 7B,C, for which two-way ANOVA, followed by the Bonferroni post-tests, was used instead. Results were considered statistically significant at *P* < 0.05.

**FIGURE 1:**
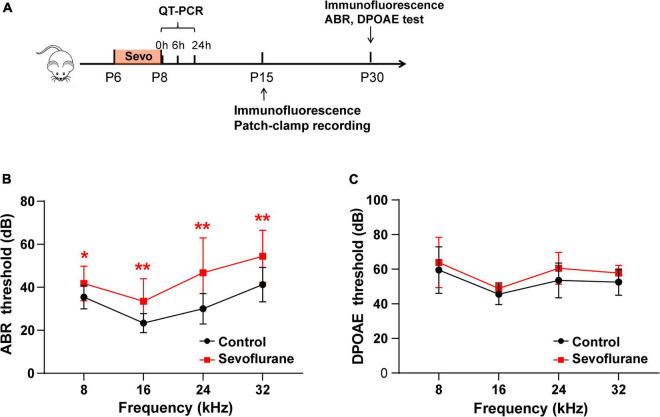
Multiple sevoflurane exposures in naïve mice impair hearing function. **(A)** Timeline of the experimental protocol. **(B)** Compared to the control group, auditory brainstem response (ABR) thresholds in the sevoflurane group are significantly elevated at 8, 16, 24, and 32 kHz (*n* = 12 in the control group, *n* = 17 in the sevoflurane group). **(C)** Distortion product otoacoustic emission (DPOAE) thresholds are comparable in both groups at different frequencies (*n* = 10 control group, *n* = 9 sevoflurane group). * *P* < 0.05, ** *P* < 0.01, unpaired Student’s *t*-test.

**FIGURE 2:**
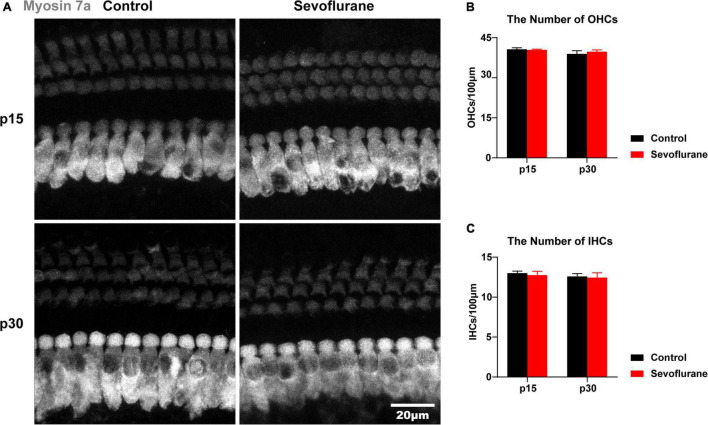
Multiple sevoflurane exposures in naïve mice do not damage hair cells. **(A)** Outer hair cells (OHCs) and inner hair cells (IHCs) identified by immunostaining for myosin VIIa (gray). The morphology and arrangement of IHCs and OHCs are comparable in the two groups on P15 and P30. **(B,C)** Quantitative data of panel **(A)** (*n* = 11 control group, *n* = 12 sevoflurane group). *P* > 0.05, unpaired Student’s *t*-test. Scale bar = 20 μm.

**FIGURE 3:**
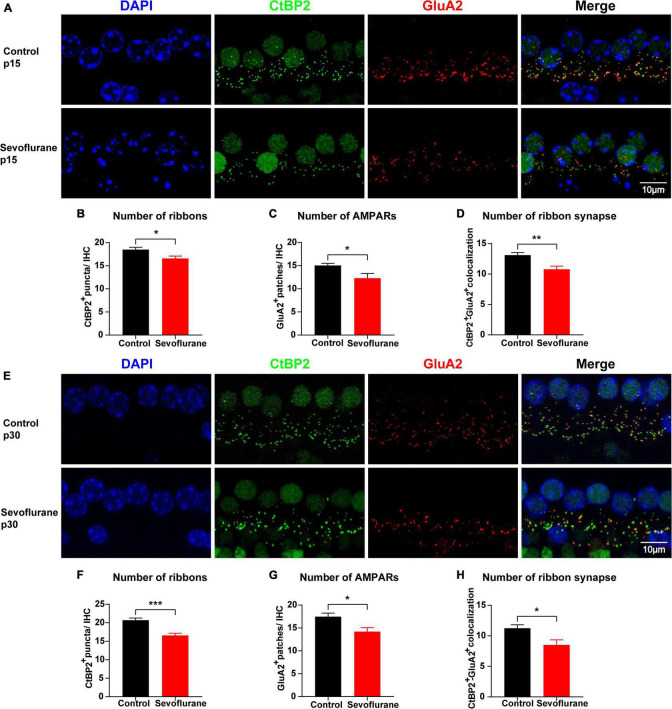
Multiple sevoflurane exposures in naïve mice cause ribbon synapse loss. **(A,E)** Pre-synaptic ribbon and post-synaptic AMPA receptors (AMPARs) labeled with CtBP2 (green) and GluA2 (red), respectively. Nuclei are labeled with DAPI (blue). After multiple sevoflurane exposures in naïve mice, ribbons, and AMPARs degenerated on P15 and P30. **(B–D)** The number of CtBP2-positive ribbons, GluA2-positive AMPAR, and ribbon synapses decreased on P15. **(E)** Ribbon synapse loss persisted on P30. **(F–H)** The number of CtBP2-positive ribbons, GluA2-positive AMPARs, and the ribbon synapse number decreased on P30 (*n* = 8 control group, *n* = 8–9 sevoflurane group). * *P* < 0.05, ** *P* < 0.01, *** *P* < 0.001, unpaired Student’s *t*-test. Scale bar = 10 μm.

**FIGURE 4:**
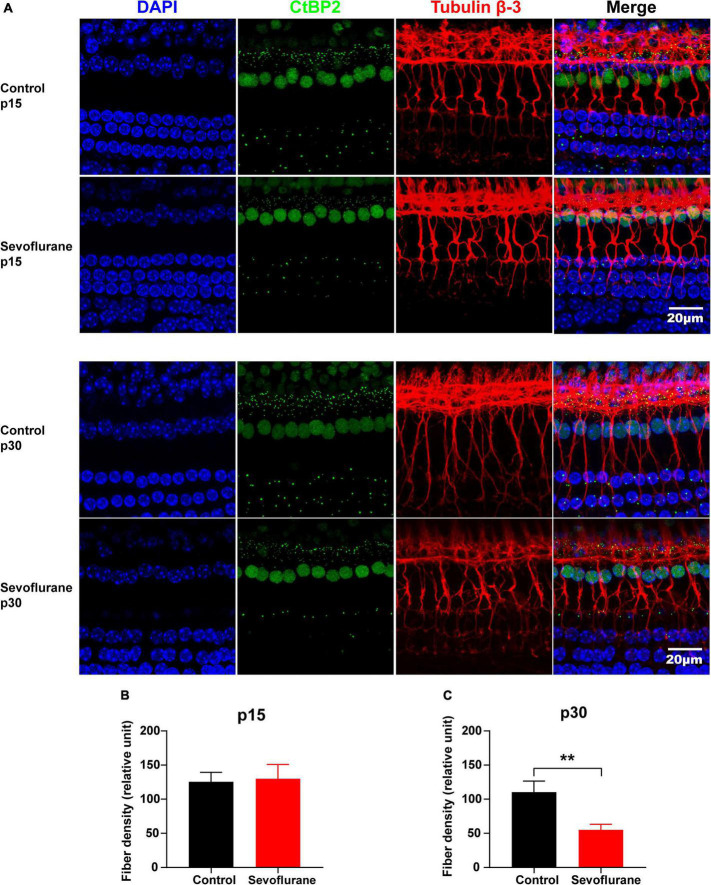
Multiple sevoflurane exposures in naïve mice lead to degeneration of spiral ganglion neuron (SGN) nerve fibers. **(A)** Pre-synaptic ribbons and nerve fibers labeled using CtBP2 (green) and tubulin β-3 (red), respectively. Nuclei are labeled with DAPI (blue). **(B,C)** The SGN peripheral fibers density on P15 **(B)** and P30 **(C)**. The density of SGN peripheral fibers is comparable in the two groups on P15. In contrast, on P30, it decreased significantly in the sevoflurane group than that in the control group (*n* = 7 control group, *n* = 8 sevoflurane group). ** *P* < 0.01, unpaired Student’s *t*-test. Scale bar = 20 μm.

**FIGURE 5:**
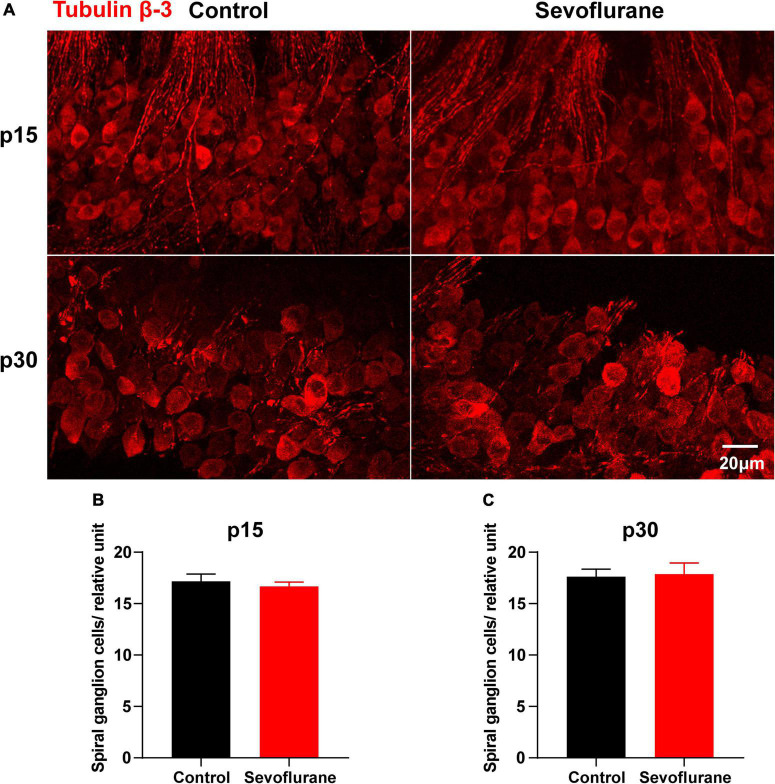
Multiple sevoflurane exposures in naïve mice do not reduce the number of spiral ganglion neurons (SGNs). **(A)** Cell bodies of SGNs identified by immunostaining for anti-tubulin β-3 (red). **(B)** SGN density is comparable in the two groups on P15. **(C)** SGN density is comparable in the two groups on P30 (*n* = 6 per group). *P* > 0.05, unpaired Student’s *t*-test. Scale bar = 20 μm.

**FIGURE 6:**
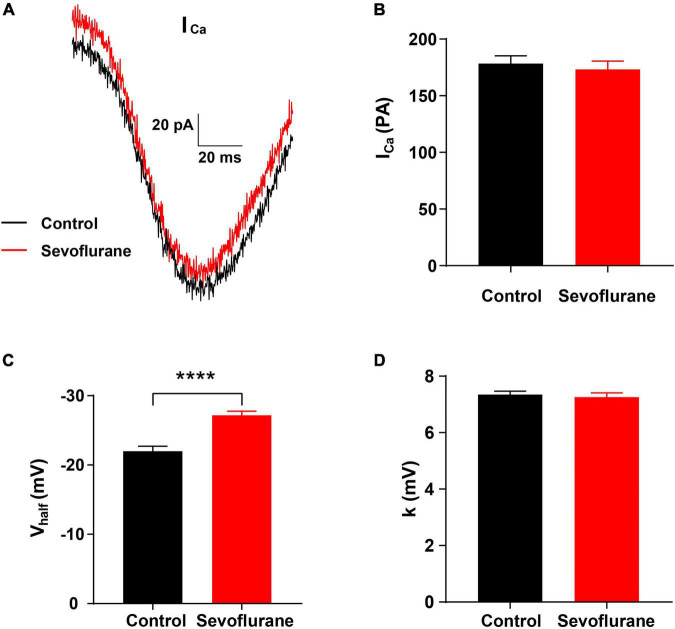
Changes in Ca^2+^ current in inner hair cells (IHCs) following sevoflurane exposure. **(A)** Representative Ca^2+^ current in IHCs in the control (black) and sevoflurane (red) groups. **(B)** After multiple sevoflurane exposures during infancy, the peak amplitude of Ca_2+_ current (I_*Ca*_) of IHCs is similar to that in the control group. **(C)** The half-activation potential (V_*half*_) is significantly more negative in the sevoflurane exposure group than that in the control group. **(D)** The slope of activation (k) is not significantly changed by multiple sevoflurane exposures in infancy (*n* = 29 control group, *n* = 51 sevoflurane group). **** *P* < 0.0001, unpaired Student’s *t*-test in panels **(B,C)**, Mann–Whitney test in panel **(D)**.

**FIGURE 7:**
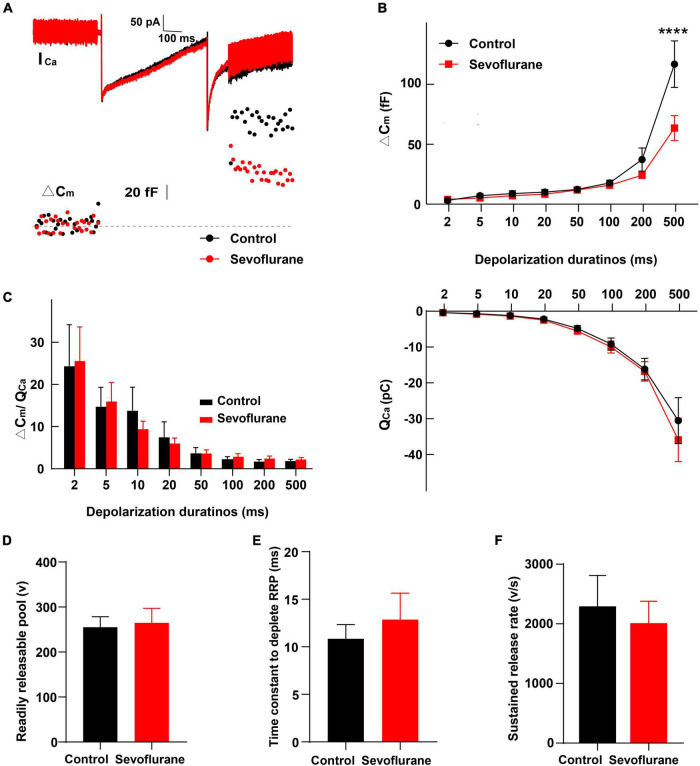
Changes in inner hair cell (IHC) exocytosis following sevoflurane exposure. **(A)** Typical Ca^2+^ currents (I_*Ca*_) and capacitance jumps (ΔC_*m*_) recorded from IHCs in the control group (black) and sevoflurane group (red). **(B)** ΔC_*m*_ and the Ca^2+^ charge (Q_*Ca*_) evoked by stimulations from 2 to 500 ms. Only for stimulations at 500 ms, ΔCm is significantly reduced following sevoflurane exposure. Q_*Ca*_ is comparable between the two groups. **(C)** No difference is observed in the ΔC_*m*_/Q_*Ca*_ ratio, a surrogate for the efficacy of Ca^2+^ to trigger exocytosis. **(D)** The readily releasable pool (RRP), **(E)** time constant to deplete RRP, and **(F)** sustained-release rate are similar between the two groups (*n* = 8–17 control group, *n* = 10–24 sevoflurane group). **** *P* < 0.0001, two-way ANOVA in panels **(B,C)**, unpaired Student’s *t*-test in panels **(D–F)**.

## Results

### Multiple Sevoflurane Exposures in Naïve Mice Elevate the Auditory Brainstem Response Thresholds Without Impacting Distortion Product Otoacoustic Emission Thresholds

According to the experimental protocol (Figure 1A), ABR and DPOAE tests were conducted at P30. In the sevoflurane group, ABR thresholds were significantly elevated when compared to those in the control group at 8 kHz (35.42 ± 1.57 vs. 41.76 ± 1.97 dB, *P* = 0.0256), 16 kHz (23.33 ± 1.28 vs. 33.53 ± 2.523 dB, *P* = 0.0012), 24 kHz (30.00 ± 2.04 vs. 46.76 ± 3.93 dB, *P* = 0.0024), and 32 kHz (41.25 ± 2.31 vs. 54.41 ± 2.94 dB, *P* = 0.0028) (Figure 1B). DPOAE thresholds indicate the functional state of OHCs. Thus, we also calculated the DPOAE thresholds at different frequencies; however, the results were similar between the two groups (*P* > 0.05; Figure 1C). Collectively, these findings indicate that OHCs are not involved in sevoflurane-induced ototoxicity.

### Multiple Sevoflurane Exposures in Naïve Mice Do Not Damage Hair Cells

Ototoxic drugs often impair hearing function by causing morphological changes in the cochlea, e.g., hair cell loss or stereocilia disruption (McFadden et al., 2004; Ding et al., 2010). Hence, we observed the morphology and number of IHCs and OHCs on P15 and P30, respectively. No differences in hair cell morphology were observed between the two groups (Figure 2A). Moreover, compared to those in the control group, no significant changes in the number of IHCs or OHCs were found in the sevoflurane group (*P* > 0.05; Figures 2B,C). These findings indicated that hair cells were spared from sevoflurane-induced hearing impairment.

### Multiple Sevoflurane Exposures in Naïve Mice Cause Ribbon Synapse Loss

To further evaluate the association between sevoflurane exposure and hearing impairment, we examined and compared the number of ribbon synapses in the two groups on P15. The number of CtBP2-positive puncta in individual IHCs was reduced by 10.3% (18.48 ± 0.52 vs. 16.58 ± 0.51 vs, *P* = 0.0208; Figures 3A,B) and that of GluA2-positive puncta was reduced by 18.3% (15.03 ± 0.46 vs. 12.28 ± 1.02, *P* = 0.0197; Figures 3A,C) in the sevoflurane group when compared to those in the control group. Similarly, the CtBP2/GluA2 double-positive puncta, presumably ribbon synapses, significantly decreased by 23.2% in the sevoflurane group compared to those in the control group (10.78 ± 0.52 vs. 13.10 ± 0.43, *P* = 0.0039; Figures 3A,D).

Ribbon synapses are capable of regeneration following ototoxic drug withdrawal (Liu et al., 2015). Thus, we quantified CtBP2, GluA2, and ribbon synapses on P30 (Figure 3E) and found their numbers in the sevoflurane group were significantly decreased by 20.0, 18.7, and 27.4%, respectively, compared with those in the control group (20.69 ± 0.58 vs. 16.56 ± 0.62, *P* = 0.0002; 17.47 ± 0.79 vs. 14.19 ± 0.88, *P* = 0.0138; 11.24 ± 0.56 vs. 8.50 ± 0.84, *P* = 0.0141, respectively; Figures 3F–H). Taken together, these findings suggest that ribbon synapse loss persists even after sevoflurane withdrawal.

### Multiple Sevoflurane Exposures in Naïve Mice Lead to the Degeneration of Spiral Ganglion Neuron Nerve Fibers

No significant difference was observed in SGN nerve fiber density between the sevoflurane and control groups in the selected area of interest on P15 (*P* > 0.05; Figures 4A,B). However, the density of SGN nerve fibers in the sevoflurane group was decreased on P30 when compared to that in the control group (55.04 ± 8.13 vs. 110.40 ± 16.23, *P* = 0.0073; Figures 4A,C).

### Multiple Sevoflurane Exposures in Naïve Mice Do Not Reduce the Number of Spiral Ganglion Neurons

The potential cause of the observed decrease in SGN nerve fiber density may be the retraction of SGN peripheral nerve fibers or the reduction of SGNs. Thus, as a proxy for possible cell death, we counted the number of SGNs on P15 and P30; however, no significant difference was observed between the control and sevoflurane groups at P15 (*P* > 0.05; Figures 5A,B). Moreover, the number of SGNs in the sevoflurane group was similar to that in the control group on P30 (*P* > 0.05; Figures 5A,C). The findings indicate that reduction in SGN peripheral nerve fiber density may result from retraction of SGN peripheral nerve processes rather than from the death of SGNs.

### The Ca^2+^ Current in Inner Hair Cells Is Altered Following Sevoflurane Exposure

To further observe the function of the IHC ribbon synapses, we conducted a whole-cell patch-clamp recording in IHCs on P15. The Ca^2+^ current responses recorded in the control and sevoflurane groups were similar (Figure 6A). Moreover, no significant changes were observed in the Ca^2+^ current (I_*Ca*_) peak amplitude of IHCs between the sevoflurane and control groups (173.30 ± 7.22 vs. 178.50 ± 6.78 pA, *P* > 0.05; Figure 6B), indicating that IHCs in both groups retained the capacity to respond to sound-trigged depolarization and evoke intracellular calcium increases.

We next compared the voltage dependence of the Ca^2+^ current by assessing the slope of activation (k) and half-activation potential (V_*half*_). The V_*half*_ of IHCs in the sevoflurane group was −27.17 ± 0.60 mV, which was significantly more negative than that of IHCs in the control group (−21.99 ± 0.70 mV, *P* < 0.0001; Figure 6C). Moreover, the k for IHCs was similar between the sevoflurane and control groups (7.26 ± 0.15 vs. 7.35 ± 0.12 mV, *P* > 0.05; Figure 6D). The findings indicate that the voltage dependence of the Ca^2+^ current is affected, and its activation becomes easier after sevoflurane exposure.

### Sevoflurane Exposure Alters Inner Hair Cell Exocytosis

Next, we compared IHC exocytosis using whole-cell membrane capacitance measurements. Exocytosis was assessed by quantifying the capacitance change before and after stimulation (ΔC_*m*_) (Figure 7A). Strong depolarizing voltage pulses to 0 mV with variant duration were applied to examine the rapid and sustained release of synaptic vesicles. The ΔC_*m*_ for different stimulation durations of 2–500 ms was calculated (von Gersdorff and Matthews, 1994; Hallermann et al., 2003). After stimulation for 2, 5,10, 20, and 50 ms, ΔC_*m*_ in the sevoflurane group was comparable to that in the control group (*P* > 0.05; Figure 7B), indicating that sevoflurane exposure did not significantly influence fast exocytosis. Similarly, exocytosis was not significantly impacted following 100 or 200 ms stimulation. However, following stimulation for 500 ms, the ΔC_*m*_ in the sevoflurane group was significantly decreased compared to that in the control group (*P* < 0.0001, Figure 7B), reflecting a reduced capacity to release neurotransmitters.

We further compared the readily releasable pool (RRP), time constant to deplete RRP, and sustained-release rate and found no difference between the two groups (Figures 7D–F). Furthermore, no significant change was observed in Ca^2+^ influx, as assessed by Ca^2+^ charge (Q_*Ca*_), between the control and sevoflurane groups (Figure 7B).

Finally, we calculated the ratio of ΔC_*m*_/Q_*Ca*,_ a surrogate for efficiency of Ca^2+^ to trigger exocytosis; however, we did not find significant differences between the two groups (*P* > 0.05, Figure 7C), implying that the reduction in ΔC_*m*_ after stimulation for 500 ms was not due to alternations in the efficiency of Ca^2+^ to trigger exocytosis.

Collectively, these results indicate that multiple exposures to sevoflurane attenuate the triggered vesicle release from IHC synaptic ribbons with unaltered Ca^2+^ influx and preserved normal Ca^2+^ influx-to-release coupling.

### Oxidative Stress in the Cochlea Is Elevated Following Multiple Sevoflurane Exposures

**FIGURE 8:**
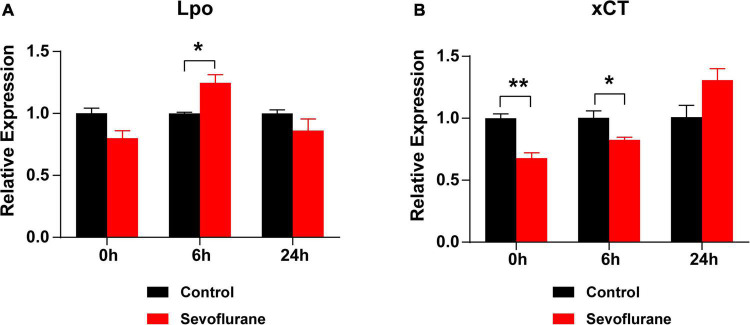
Oxidative stress is elevated after sevoflurane exposure. **(A)** mRNA expression of *Lpo* is upregulated at 6 h, rather than at 0 or 24 h, in the sevoflurane group compared to that in the control group. **(B)** The mRNA expression of *xCT* is downregulated at 0 and 6 h in the sevoflurane group. No difference is observed between the control and sevoflurane groups at 24 h (*n* = 6 per group). * *P* < 0.05, ** *P* < 0.01, unpaired Student’s *t*-test.

We hypothesized that oxidative stress plays a key role in sevoflurane-induced ototoxicity. To test this hypothesis, the mRNA expression levels of *Lpo* and *xCT*, two oxidative stress-related genes, were compared at 0, 6, and 24 h after sevoflurane exposure. At 0 h, the expression of *Lpo* was comparable between the two groups (*P* > 0.05), whereas *xCT* expression was significantly lower in the sevoflurane group (*P* = 0.0045). At 6 h, *Lpo* was significantly upregulated (*P* = 0.0219), whereas *xCT* remained downregulated (*P* = 0.0425) in the sevoflurane group. At 24 h, *Lpo* and *xCT* expression did not differ between the two groups (*P* > 0.05 for both; Figures 8A,B). Furthermore, administration of the antioxidant agent NAC before sevoflurane exposure successfully reversed the hearing impairment caused by sevoflurane exposure (*P* = 0.0290; Supplementary Figure 1). These findings suggest that elevated oxidative stress contributes to sevoflurane-induced ototoxicity in the cochlea.

## Discussion

The ABR threshold is typically used to evaluate hearing function (Sun et al., 2014); therefore, the ABR test is commonly used to diagnose diseases of the inner ear, cerebellopontine angle, and central auditory pathways (Ruth and Lambert, 1991). In the current study, the ABR thresholds of mice in the sevoflurane group were significantly elevated, suggesting hearing impairment after multiple sevoflurane exposures in naïve mice.

Damage or loss of hair cells is a common root cause for different types of hearing impairment. DPOAE thresholds indicate the functional state of OHCs. In the current study, neither the morphology and number of hair cells nor the DPOAE thresholds in the sevoflurane group were significantly different from those in the control group. The findings indicate that sevoflurane-induced ototoxicity is mediated by mechanisms that do not involve hair cells.

In the auditory pathway, ribbon synapses serve as the first afferent synaptic connection and play a significant role in accurate sound transmission (Grant et al., 2010; Moser and Vogl, 2016). Ribbon synapses are vulnerable to trauma. Many toxic agents, as well as noise-induced injury, can result in ribbon synapse degeneration, leading to hearing impairment (ShuNa et al., 2009; Liu et al., 2013). Fujimoto and Yamasoba (2014) reported that ototoxic aminoglycoside stimuli primarily target the cochlear IHC ribbon synapse. Nevertheless, maintaining the ribbon synapse number *via* fibroblast growth factor 22 (FGF22) is promising to protect against hearing impairment induced by gentamycin (Li et al., 2016). Moreover, Sun et al. (2019) found that sevoflurane exposure during infancy was associated with synaptic ultrastructural impairments in the hippocampus and temporary spatial working memory deficits. Our previous study reported ribbon synapse loss and hearing loss after *in utero* sevoflurane exposure in mice (Yuan et al., 2020). In this study, we found a 10–25% loss in CtBP2, GluA2, and the paired CtBP2/GluA2 double-positive patches on both P15 and P30, which may account for the observed changes in ABR.

We also compared the function of the IHC ribbon synapse after sevoflurane exposure through patch-clamp recordings. Ca^2+^ influx by voltage-gated Ca^2+^ channels is essential in transmitting auditory signals in IHC ribbon synapses (Zampini et al., 2013). However, the amplitude of the Ca^2+^ current was similar between the two groups. Therefore, we further compared the voltage dependence of the Ca^2+^ current between the two groups with V_*half*_ and k and found that V_*half*_ was markedly more negative in the sevoflurane group, indicating an alteration in the voltage dependence of the Ca^2+^ current after multiple sevoflurane exposures in infants. Accordingly, the high temporal precision of acoustic signal encoding relies on the sustained release of synaptic vesicles from IHCs (Moser and Beutner, 2000; Johnson et al., 2005; Ruel et al., 2008). IHCs are capable of fast and sustained exocytosis of synaptic vesicle. Rapid exocytosis, lasting less than 50 ms (Graydon et al., 2011), represents the release of an RRP of synaptic vesicles. Sustained exocytosis, lasting up to a few seconds, reflects the highly efficient recycling of synaptic vesicles (Meyer et al., 2009). In the present study, only sustained exocytosis at 500 ms was reduced on P15 after sevoflurane exposure, suggesting alteration of the slow secretory component induced by sevoflurane. Loss of ribbon synapses may partially account for the altered voltage dependence of the Ca^2+^ current and the stimulation duration-dependent reduction of exocytosis.

Spiral ganglion neuron nerve fibers are responsible for transmitting signals from cochlear IHCs to the brainstem. For instance, the rat cochlea is innervated by more than 19,000 nerve fibers (Dannhof and Bruns, 1993), and ∼95% of the cochlear nerve fibers form synaptic connections only with IHCs (Spoendlin, 1972). Each auditory nerve fiber only receives signals from one IHC through one ribbon synapse only (Spoendlin, 1969; Liberman, 1980), whereas each hair cell is connected to 10–30 auditory nerve fibers (Bohne et al., 1982; Liberman et al., 1990; Stamataki et al., 2006). Studies have shown that moderate acoustic overexposure causes reversible threshold elevation and irreversible degeneration of auditory nerve fibers (Furman et al., 2013; Liberman et al., 2015). Moreover, cochlear nerve terminal swelling and corresponding degeneration of SGN nerve terminals are shown to be associated with a loss of synaptic ribbons (Kujawa and Liberman, 2009). Research on electrical stimulation-induced hearing impairment also found a decrease in the density of SGN peripheral fibers, accompanied by ribbon synapse loss (Liang et al., 2019). Consistent with these studies, our study demonstrated that the SGN nerve fiber density decreased on P30 in the sevoflurane group. In contrast, this type of degeneration was not detectable on P15, possibly due to ribbon synapses, which contact auditory afferent fibers, being more vulnerable to trauma than nerve fibers. It has been shown that upon exposure to noisy environments, the hair cells and hearing thresholds are preserved; however, the IHC-SGN synapses are immediately eliminated, which reduces the supra-threshold responses (Kujawa and Liberman, 2009). However, it remains unknown whether the loss of ribbon synapses induced by sevoflurane can cause secondary restriction of SGN nerve fibers, leading to the delayed decrease in SGN nerve fiber density within the area near the ribbon synapse.

Studies on animals exposed to noise or ototoxic agents have reported that even though hair cell loss can be detected within hours (Webster and Webster, 1978; Lawner et al., 1997; Bohne and Harding, 2000; Wang et al., 2002; Suzuki et al., 2008), the loss of spiral ganglion cells is typically delayed and cannot be detected for weeks to months after insult (Johnsson, 1974; Webster and Webster, 1978; Miller et al., 1997; Sugawara et al., 2005). It has also been reported that SGN degeneration is delayed months after trauma even though hair cells were intact (Kujawa and Liberman, 2006). Heeringa et al. (2016) also observed that reduced ipsilateral SGN densities were only detectable with increased ipsilateral ABR threshold in mice sacrificed 4 weeks after intra-cochlear kanamycin injections but not in those sacrificed at 3 weeks after the injections. In addition, neonatal sevoflurane anesthesia decreases the expression of post-synaptic density protein-95 (PSD-95) in the brain in a time-dependent manner without neuronal loss (Wang et al., 2013). Other studies have considered SGN degeneration as a secondary issue to hair cell loss. In some of these studies, SGN loss was only observed in regions in which OHCs were severely destroyed, and a significant portion of IHCs, as well as most OHCs, died (Kiang et al., 1976; Liberman and Kiang, 1978; Duan et al., 2000; McFadden et al., 2004). In our study, IHCs and OHCs were intact on P15 and P30, which may explain why the number of SGNs did not decrease throughout the study period. Therefore, a prolonged study duration may help observe the exact changes in SGN.

Numerous studies have reported that oxidative stress plays a significant role in hearing impairment following exposure to noise or toxic agents, or aging (Fujimoto and Yamasoba, 2014; Wang and Puel, 2018). Excessive accumulation of intracellular reactive oxygen species has been suggested as a common and major pathology of cisplatin- and aminoglycoside antibiotic-induced ototoxicity (Thomas Dickey et al., 2004). We found that *Lpo*, a prooxidant gene encoding lactoperoxidase that contributes to lipid peroxidation and oxidative stress (Ghibaudi et al., 2000), was significantly upregulated after sevoflurane exposure. However, *xCT*, a typical antioxidant gene (Sims et al., 2012), was markedly downregulated. These findings indicate that acute oxidative stress caused by sevoflurane may have long-term effects on the peripheral auditory nerve system. NAC is an acetylated cysteine with antioxidant properties that can neutralize free radicals before they cause damage to cells and increase cysteine/GSH levels in cells (Samuni et al., 2013). Moreover, NAC protects against cisplatin-induced hearing loss in rats (Thomas Dickey et al., 2004). It also protects the central auditory system by reducing synaptic loss in the dorsal cochlear nucleus (Du et al., 2012). In our study, NAC administration successfully decreased ABR thresholds, suggesting the protective effect of NAC against sevoflurane-induced ototoxicity.

Our study has certain limitations. First, we did not compare the amplitude of ABR wave I, which represents the function of ribbon synapses (Tan et al., 2017). Second, we only compared synaptic ribbons and glutamate (GluA2) receptors in terms of the number per IHC. Previous studies have shown that the volume and position of ribbons and AMPAR patches are also affected in noise-induced hearing impairment models (Liberman et al., 2015; Paquette et al., 2016; Kim et al., 2019). Therefore, further studies regarding the volume and position of ribbons and AMPAR patches are warranted using the sevoflurane exposure model. Third, we did not compare the SGN nerve fibers in terms of length and diameter; changes in these critical properties should be detected in future studies.

In conclusion, the findings demonstrate that multiple sevoflurane exposures during the neonatal period cause hearing impairment in adult mice. The study also showed that elevated oxidative stress led to ribbon synapses impairment and SGN nerve fibers degeneration, and the interventions of antioxidants alleviated the sevoflurane-induced hearing impairment. Overall, the study indicates that the developing peripheral auditory nervous system is vulnerable to environmental insult, including multiple exposures to certain anesthetics, and raises concerns regarding the safety of anesthesia administration to children and its potential adverse post-operative outcomes. It suggests further studies to develop strategies to deal with such adversities of anesthetics in children.

## Data Availability Statement

The raw data supporting the conclusions of this article will be made available by the authors, without undue reservation.

## Ethics Statement

The animal study was reviewed and approved by the Animal Care and Use Committee of Fudan University.

## Author Contributions

XS and G-LL designed the study. YL, HY, XZ, and WL conducted the study. YL, HY, XS, and G-LL analyzed the data and wrote the manuscript. All authors contributed to data analysis, drafting, and revising the manuscript and agreed to be accountable for all aspects of the work.

## Conflict of Interest

The authors declare that the research was conducted in the absence of any commercial or financial relationships that could be construed as a potential conflict of interest.

## Publisher’s Note

All claims expressed in this article are solely those of the authors and do not necessarily represent those of their affiliated organizations, or those of the publisher, the editors and the reviewers. Any product that may be evaluated in this article, or claim that may be made by its manufacturer, is not guaranteed or endorsed by the publisher.
