# Supplementary material for: Multiple Sevoflurane Exposures During the Neonatal Period Cause Hearing Impairment and Loss of Hair Cell Ribbon Synapses in Adult Mice
Source: Front Neurosci. 2022 Jul 14;16:945277. doi: 10.3389/fnins.2022.945277 (PMC9329801; doi:10.3389/fnins.2022.945277)
Supplement: Supplementary file 1 [file Image_1.pdf]

Supplementary Material

Figure. S1

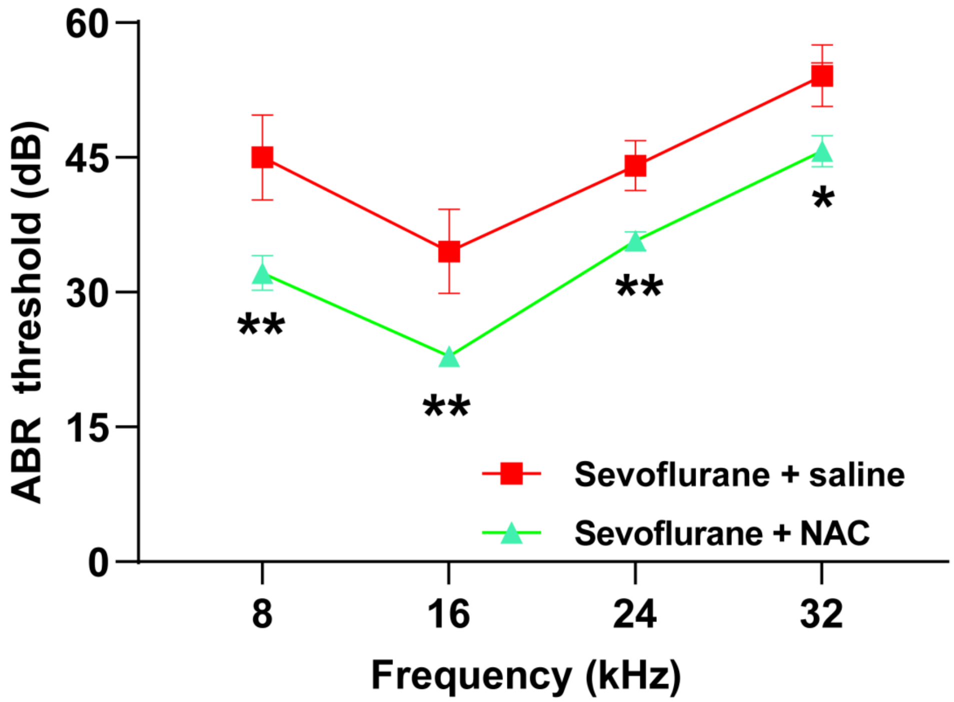

Figure S1 *N*-acetylcysteine (NAC) treatment rescued sevoflurane-induced hearing impairment.

Compared to that in the sevoflurane group, ABR thresholds in mice receiving NAC + sevoflurane are significantly decreased at four frequencies. (n = 11 in the sevoflurane + saline group, n = 14 in the sevoflurane + NAC group). * $P < 0.05$, ** $P < 0.01$, Mann–Whitney test at 8 and 16 kHz, unpaired Student's *t*-test at 24 and 32 kHz.
